# HIV-1 infection facilitates Alzheimer’s disease pathology in humanized APP knock-in immunodeficient mice

**DOI:** 10.1515/nipt-2024-0018

**Published:** 2025-02-10

**Authors:** Shaurav Bhattarai, Rana Kadry, Pravin Yeapuri, Yaman Lu, Emma G. Foster, Chen Zhang, Prasanta Dash, Larisa Y. Poluektova, Santhi Gorantla, R. Lee Mosley, Howard E. Gendelman

**Affiliations:** Department of Pharmacology and Experimental Neuroscience, College of Medicine, University of Nebraska Medical Science, Omaha, NE, USA

**Keywords:** HIV-1, Alzheimer’s disease, mouse model, humanized mice, immune deficient, amyloid beta

## Abstract

**Objectives:**

Amyloid-β (Aβ) plaque deposition in the brain is a principal pathological feature of both Alzheimer’s disease (AD) and progressive human immunodeficiency virus type one (HIV-1) infection. Both enable Aβ assembly and Aβ protein aggregation. The potential link between HIV-1 and AD remains uncertain, supporting the need for a reliable animal model. HIV-1 is tropic and pathogenic for humans. It does not replicate in mice. The restricted species tropism has slowed progress in basic research activities. The current study seeks to correct animal model limitations.

**Methods:**

We created an AD mouse to address the need to develop an small animal model that allows studies of viral infection by making a knock-in (KI) with the human amyloid precursor protein (APP)^KM670,671NL^ Swedish mutation to the mouse genome. The resulting founder mice were crossed with immunodeficient NOG (NOD. Cg-*Prkdc*^*scid*^*Il2rg*^*tm1Sug*^Tg(CMV*-IL-34*)*1*/Jic) to generate NOG/APP^KM670,671NL^/IL-34 (NAIL) mice. The mice were reconstituted with human hematopoietic stem cells to generate NAIL mice with functional adaptive and innate human immune systems. Four-month-old, humanized NAIL mice were infected with HIV-1_ADA_, a macrophage-tropic viral strain then evaluated for viral infection and AD pathology.

**Results:**

Productive HIV-1 infection was confirmed by plasma HIV-1 RNA levels in infected NAIL mice. The viral load increased by tenfold between day 10 and day 25 post-infection. By day 25, viral DNA confirmed the establishment of HIV-1 reservoirs in CD45+ cells from the immune tissues of infected NAIL mice. Additionally, p24 measurements in lymphoid and brain tissues of NAIL mice validated productive HIV-1 infection. Amyloid burden from infected NAIL mice was increased. Immunofluorescence staining revealed co-localization of Aβ fibrils and HLA-DR^+^ microglia in infected NAIL mice.

**Conclusions:**

These results highlight the AD-HIV model’s unique pathobiological and infectious features where the viral and immune responses can now be measured.

## Introduction

Antiretroviral therapy (ART) has increased the lifespan and disease morbidities of those infected with human immunodeficiency virus type one (HIV-1). Currently, 38 million people worldwide are living with HIV-1. Despite effective viral suppression, up to 50 % of infected persons succumb to a range of neurological, motor, and cognitive deficits collectively termed HIV-associated neurocognitive disorder (HAND) [1], [2], [3], [4]. Moreover, age-associated morbidities that include Alzheimer’s disease (AD) are on the rise in those HIV-1-infected persons [4], [5].

In aged populations, AD is on a meteoric rise in incidence and prevalence [6]. Today it is the most common form of dementia and is characterized by crippling memory, behavioral, and cognitive declines. The disease is linked to advancing age, deposition of misfolded protein aggregates, and inflammation [7], [8]. Disease mortality has doubled from 2000 to 2019, now accounting for nearly one out of three deaths in the United States’ elderly population [6].

An overlap in disease pathology exists between HIV-1 infection and AD, as both conditions drive neuroinflammation, synaptic dysfunction, and neuronal damage [9], [10], [11]. Monocytes, brain macrophages, and microglia are the principal targets for HIV infection in the brain [12]. The viral infection leads to neuroinflammation and neuronal dysfunction with increases in chemokine expression and types I interferons (IFNs) and IFN-stimulated genes that include but are not limited to MX1, ISG15, and ISG20 [13]. Production of proinflammatory factors persists even after the addition of antiretroviral therapy (ART) [14]. AD is historically characterized by the deposition of misfolded amyloid β (Aβ) plaques and tau neurofibrillary tangles. Still, chronic immune activation has been an essential disease-associated contribution over the last decade [15]. Chronic immune activation drives neuroinflammation in various neurodegenerative and infectious diseases, leading to cognitive impairments, as observed in AD and HIV-1 infection [11]. Aβ plaques, which are typically associated with AD, have been observed in the brains of HIV-1-infected individuals, suggesting a plausible pathological link between the two diseases [16], [17], [18]. While the exact disease mechanisms are not fully understood, interactions between viral infection, CD4+ T cells, misfolded protein aggregation, and ART-linked toxicities are assumed to accelerate AD [19], [20]. Furthermore, persistent viral infection, variable ART brain penetration, and disruption of immune homeostasis in the setting of HAND could contribute to progressive neurodegenerative disease [21], [22], [23].

Currently, due to the species restriction of HIV-1 infection, no AD mouse models are available that permit studies of AD during HIV-1 infection [24], [25], [26], [27]. Thus, an immediate need to understand the relatedness of AD in the setting of age-related viral infection and other co-morbidities is warranted. These viral and immune interactions can affect the onset and progression of neurodegenerative diseases. Previous studies have been limited to evaluations of common viral proteins such as HIV-1gp120 and Tat used in existing transgenic models or by reconstituting immune-deficient animals with adult human lymphocytes [28], [29]. In both instances, using a mouse model carrying murine amyloid precursor protein (APP) contains some limitations, as differences between human and murine APP complicate the clinical relevance of the findings [28], [30]. Therefore, this highlights the need to develop a suitable rodent model to examine the interactions between human pathological protein Aβ and HIV-1.

We developed a novel AD mouse model using CRISPR-Cas9 technology to knock in human mutant *APP* loci and overcome these obstacles [31]. These mice were created on an immunocompromised NOG (NOD Cg-*Prkdc*^*scid*^*Il2rg*^*tm1Sug*^/JicTac) background to facilitate studies of HIV-1 infection. The mouse model was generated by genetically modifying the mouse amyloid precursor protein (*APP*) gene to encode the human APP Swedish mutation by knocking in (KI) the human APP^KM670,671NL^ mutations (NOG/APP or NA mice). To generate human immune reconstitution, NA mice were crossed with NOD. Cg-*Prkdc*^*scid*^*Il2rg*^*tm1Sug*^Tg(CMV-*IL34*)1/Jic (NOG/Tg(CMV-*IL34*)) mice transgenic for human *IL34* [32]. This allowed the investigation of human microglial responses by creating NOG/APP^KM670,671NL/^/*IL-34 *(NAIL) mice. Transplantation of NAIL mice with human hematopoietic stem cells (HSCs) reconstituted both human innate and adaptive cell networks that allow the study of innate human-like microglia in the brain as well as peripheral innate and adaptive immunity in lymphoid tissues and blood in the context of HIV-1 infection and AD.

In this novel model, NAIL mice express familial AD mutations driven by the endogenous promoter. These mice are developed in an immunocompromised NOG background to allow human-immune reconstitution and support permissive HIV-1 infection. This new humanized mouse model could emerge as an essential tool for studying the pathological process of AD, particularly in the context of progressive HIV-1 infection. The characteristics of neuronal and glial changes that occur with disease progression are known to shape both HIV and AD pathology through complex immunoregulatory mechanisms [33], [34]. Gene mutations in myeloid cells affect immunological pathways and alter the risks for disease progression in both HIV and AD [35], [36], [37]. Metabolic abnormalities, impaired meningeal lymphatics, and autoimmunity are also linked to both diseases. Therefore, this new mouse model will be helpful in the study of the complex interplay between immunity and CNS disease.

## Methods

### Study approvals

All animal procedures in these studies followed the Institutional Animal Care and Use Committee (IACUC) guidelines approved protocols at the University of Nebraska Medical Center (UNMC).

### Development and generation of NAIL mice

**Figure 1:**
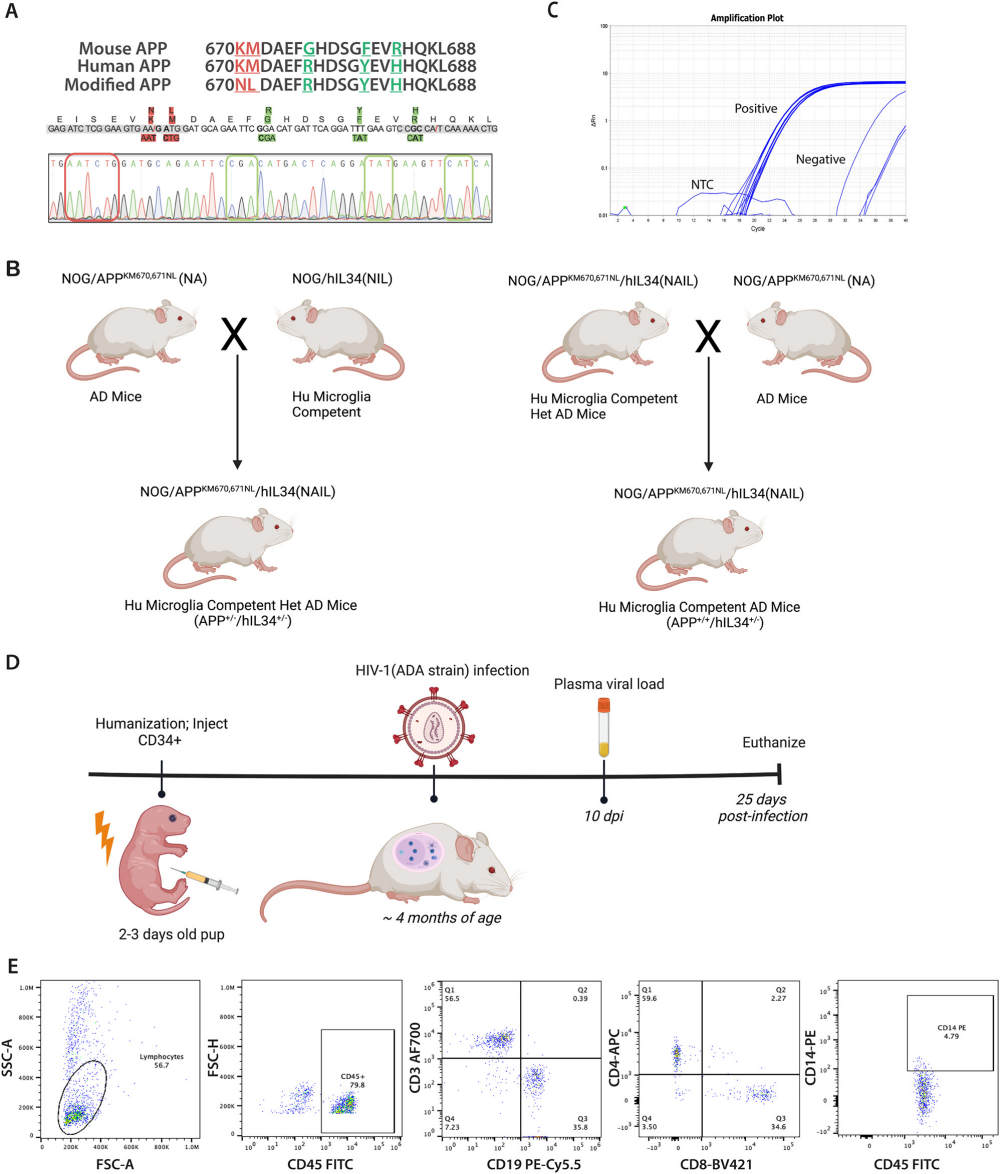
Development of AD humanized mouse knock in of a APP (Swe) mutation. (A) Nucleotide and amino acid substitutions in exon 16 provided to introduce the APP Swedish mutation (APP^KM670,671NL^) (red highlighted) and humanize the mouse *APP *exon 16 (green highlighted) as shown by Sanger sequencing. This confirmed replacement of pathological (red box) and humanized (green box) nucleotides in mouse *APP* gene. (B) Developmental scheme for generating NAIL mice showing (NOD Cg-*Prkdc*^*scid*^*Il2rg*^*tm1Sug*^/JicTac) (NOG) with knocked in APP^KM670,671NL^ (NA) mice. Homozygous NA mice were crossed with NOD Cg-*Prkdc*^*scid*^*Il2rg*^*tm1Sug*^Tg(CMV*-IL34*)*1*/Jic that are competent for the development of human-like microglia (Hu Microglia) to generated the NAIL mice. First-generation NAIL mice carry heterozygous human *APP* and human *IL34* genes. Homozygous NA mice were backcrossed with heterozygous NAIL mice to generate second-generation NAIL mice that carry homozygous human *APP* and heterozygous human *IL34* genes with competency for permissive human-like microglia development. (C) qPCR analyses confirmed the presence of the human *IL34* gene in NAIL mice. (D) Animal study design showing the timeline of experimental interventions. Neonatal NAIL mice were engrafted with HSCs at 2–3 days after birth and were monitored for human CD45+ immune cell reconstitution for 3 months by peripheral blood flow cytometry. At approximately four months of age, human immune reconstituted NAIL mice were infected with 10^4^ TCID_50_ of HIV-1_ADA_ and monitored for infection at 10- and 25 days post-infection. Mice were sacrificed at 25 days following infection. (E) Representative flow cytometric gating and frequencies of human immune cells in peripheral blood of 3-month-old NAIL mice. The presence of human CD45+ immune cells, human T cells (CD3+, CD4+, and CD8+), human B cells (CD19+), and human monocyte/macrophages (CD14+) in NAIL mice confirmed human immune reconstitution of NAIL mice before HIV-1 infection.

NAIL mice were generated on a NOD. Cg-*Prkdc*^*scid*^*Il2r*^*gtm1Sug*^/JicTac (NOG) (Taconic, Germantown, NY) background carrying a knocked-in APP^KM670,671NL^ and transgene for *IL34* (NOG/APP^KM670,671NL^/[*IL34*]) (NAIL mice) [31]. Briefly, the human gene containing a familial AD mutation was knocked in by substituting five nucleotides at exon 16 of mouse *APP* (Figure 1A). The humanized genomic sequences of familial AD mutations were introduced into fertilized eggs of pregnant NOG mice through microinjection. Appropriate guide RNAs were used to remove mouse exons, and the mouse donor DNA was replaced with human mutations through DNA repair. The eggs were then transplanted into pseudo-pregnant recipients, and the offspring were genotyped through gene-specific RT-PCR and Sanger sequencing. Sanger sequencing was used to confirm the knock-in of humanized *APP* sequences. Ear tissue was collected at weaning and digested using an alkaline lysis buffer 0.4 M NaOH and 0.2 mM ethylene diamine tetraacetic acid (EDTA). Samples were heated at 95 °C for 1 h until fully digested. Samples were then neutralized with a neutralization buffer (40 mM Tris-HCl) and centrifuged at 10,000 *g* for 5 min. Supernatants were used for DNA amplification. Polymerase chain reaction (PCR) was performed to amplify the gene of interest (*APP*) using GoTaq Hot Start Green Master Mix (cat. M5123, Promega, Madison, WI) and forward and reverse primers specific to each gene (*APP* for: GGCGGTCACACTAACGGATG, APP rev: GACACAGGACAAGCCACGAG). PCR products were confirmed by gel electrophoresis on a 1 % agarose gel, then purified using the ZR DNA Sequencing Clean-up Kit (cat. D4051, Zymo Research, Irvine, CA). Following purification, samples were mixed with the forward primer for the gene of interest and submitted to Azenta Life Sciences (Burlington, MA) for sequencing. SnapGene Viewer was used to visualize nucleotide sequences and identify mutations to determine whether mice were heterozygous or homozygous for humanized *APP* [31].

APP-KI mice were crossed with another immunodeficient NOG background mice containing human *IL34 *transgene (NOD Cg-Prkdc^scid^Il2rg^tm1Sug^Tg(CMV-IL34)1/Jic) (referred to as hIL34/NOG) to generate NAIL mice (Figure 1B). Since mice are on an immunocompromised NOG background, they allow the reconstitution of human adaptive immune cells. Additionally, human IL34 transgene enables the development of human microglia in the brain after the engraftment of hematopoietic stem cells. The presence of transgenic human *IL34* by NAIL mice was compared to background NOG mice using a human *IL34* gene-specific primer (Hs01050926_m1). One-to two-day-old neonates were toe-clipped before human hematopoietic stem cell reconstitution to perform qPCR to confirm the presence of the *hIL-34* gene in NAIL mice. We used the following real-time PCR thermal cycle setting: 50 °C for 2 min, 95 °C for 15 s, 40 cycles of denaturation at 95 °C for 15 s, and annealing at 60 °C for 1 min. We used GAPDH as the housekeeping gene. A cycle-threshold (CT) value of less than 30 (CT<30) was considered positive for the presence of the human *IL34* gene in NAIL mice [32].

### Reconstitution of the human immune system in NAIL mice

NAIL mice, 2–3 day-old neonates, were irradiated with 1 Gy (RS 2000 X-ray Irradiator, Rad Source Technologies, Inc., Suwanee, GA, USA). Four hours post-irradiation, neonates received an intrahepatic injection of 10^5^ CD34+ hematopoietic stem cells (HSCs) derived from human cord blood (Lonza Biosciences, Rockland, ME). The purity of the CD34+ HSC (>90 % purity) was confirmed by the supplier (Lonza Biosciences) using flow cytometry. After 3 months of CD34+ cell engraftment, mice were bled from the submandibular vein for flow cytometry to confirm the reconstitution of human peripheral immune cells [38]. Six reconstituted animals were used in the study.

### Flow cytometry of human immune cell populations from NAIL mice

Blood samples were collected in EDTA-containing tubes (BD Microtainer, Franklin Lakes, NJ, USA) and centrifuged at 350 *g* for 8 min. Blood cells were resuspended in a fluorescence-activated cell sorting (FACS) buffer 2 % fetal bovine serum (FBS) and 0.1 % sodium azide in phosphate-buffered saline (PBS), then incubated for 30 min at 4 °C with a cocktail of antibodies against human immune cell markers that included fluorescein isothiocyanate (FITC)-anti-CD45, Alexa Fluor 700 (AF700)-anti-CD3, phycoerythrin-cyanin 5 (PE-Cy5)-anti-CD19, allophycocyanin (APC)-anti-CD4, Brilliant Violet 421 (BV421)-anti-CD8, and phycoerythrin (PE)-anti-CD14. All antibodies and isotype controls were obtained from BD Biosciences (San Jose, CA). Red blood cells were lysed by FACS lysing solution (BD Biosciences). Stained cells were washed with FACS buffer and fixed with 2 % paraformaldehyde [39]. Data acquisition was performed with acquisition software FACS Diva v6 (BD Biosciences) interfaced with a BD LSR2 flow cytometer (BD Biosciences), and data were analyzed using FLOWJO analysis software v10.2 (Tree Star, Ashland, OR). Gates were assigned according to the appropriate control population.

### HIV-1 infection, viral load measurement, and tissue procurement

Four-month-old HSC-engrafted NAIL mice were infected intraperitoneally with 10^4^ TCID_50_ of macrophage-tropic HIV-1_ADA_ strain. Mice that received sterile saline injection served as controls. Three HSC-reconstituted NAIL mice were infected with HIV-1, and three saline-injected HSC-reconstituted NAIL mice served as controls. Mice were bled 10 days after the infection to evaluate plasma viral load using the automated COBAS Ampliprep V2.0/Taqman-48 system (Roche, Boston, MA) [40]. Animals were euthanized 25 days post-infection, and tissues were collected for further analysis. Under terminal pentobarbital anesthesia (Fatal Plus), mice were perfused via cardiac puncture with PBS, and the brain was carefully removed. The right hemisphere was separated and placed in 4 % paraformaldehyde (Sigma-Aldrich, St. Louis, MO) in PBS. The left hemisphere was dissected, and the cortices and hippocampi were flash-frozen and stored at −80 °C for biochemical analyses. Tissue viral load in the brain and spleen was measured [39].

### Aβ ELISA

Frozen mouse cortices were homogenized in 50 mM Tris-HCL (pH 7.6) containing 150 mM NaCl and a protease inhibitor. Lysates were centrifuged at 20,000 *g* for 60 min at 4 °C, and the supernatants were collected to assess soluble Aβ_42_. To assess insoluble Aβ_42_, pellets were dissolved using 6M guanidine-HCL and centrifuged at room temperature at the same speed and time. Following the manufacturer’s protocol, Aβ_42_ concentrations in the supernatants were quantified using an ELISA kit (Quantikine^®^ ELISA, cat. DAB142, R&D Systems, Minneapolis, MN) [41].

### Immunohistochemical (IHC) and immunofluorescence (IF) staining

After transcardial perfusion, the right hemispheres of mouse brains were immersed in freshly depolymerized 4 % paraformaldehyde in PBS for 24 h at 4 °C. Brains were then transferred to 70 % ethanol until ready for processing. Brains were processed overnight in an Eperdia™ STP 120 Spin tissue processor (Thermo Fisher Scientific, Waltham, MA) and embedded in paraffin blocks. Sections of 5 μm thick were collected directly onto slides for IHC staining. IHC slides were deparaffinized and antigen retrieval was performed using Trilogy (cat. 922p-07, Sigma-Aldrich)^®^ reagent as previously described [32]. Brain sections were blocked with 10 % normal goat serum to prevent non-specific staining. Blocked brain sections were incubated overnight at 4 °C with primary mouse monoclonal antibody anti-human leucocyte antigen DR (HLA-DR antibody; cat.NB600-989, Novus Biologicals, Centennial, CO), mouse monoclonal antibody anti-HIV-1 p24 (sc-65918, Santa Cruz, Santa Cruz, CA), rabbit anti-ionized calcium-binding adaptor molecule 1 (IBA1, cat, 013-27691, Fujifilm, Valhalla, NY) or mouse anti-human Aβ (6E10, cat. 803014, Biolegend, San Diego, CA). Following washing, sections were incubated with horseradish peroxidase (HRP)-conjugated anti-mouse/rabbit secondary antibody (cat. GTX83398, GeneTex, Irvine, CA) for 1 h at room temperature. Sections were washed and reacted with hydrogen peroxide substrate and 3,3′-diaminobenzidine (DAB) as chromogen (cat. D4293, Millipore, Sigma, Burlington, MA). Sections were counterstained with hematoxylin (cat. 10,015-074, VWR, Radnor, PA). Slides were imaged using a Zeiss Axioscan 7 whole slide imaging system.

For IF staining, cryopreserved left hemisphere brain tissue was processed as described previously [41]. Briefly, 30 μm thick free-floating brain sections were blocked using 10 % normal goat serum. Following blocking, sections were incubated with mouse monoclonal antibody anti-HLA-DR (Novus, cat. NB600-989) and rabbit antibody anti-Aβ fibril OC (cat. AB2286, Millipore, Sigma) overnight at 4 °C. Following washing, tissue was incubated with Alexa Fluor 568-conjugated goat anti-mouse IgG (cat. A11004, Invitrogen. Carlsbad, CA) and Alexa Fluor 488 goat anti-rabbit IgG (cat. A11008, Invitrogen) secondary antibody for 1 h at room temperature. Images were obtained using a Zeiss Axioscan 7 whole slide imaging system.

### Statistical analysis

Data were presented as mean values ± standard error of the mean (SEM). Statistical comparisons of the means were performed with Student’s *t*-test (GraphPad Software v9.0, San Diego, CA). A p-value of less than 0.05 was considered statistically significant.

## Results

### Development of single knock in APP(Swe) mutation NOG (NA) mice

The genomic DNA sequences of mouse *APP* and human *APP* genes were determined from the NCBI database, and sequences for the Aβ_1–42_ peptide encoded by the sequences of exon 16 were analyzed and compared. The two mutations (K670N and M671L) were knocked into exon 16 using CRISPR/Cas9 technology to develop APP knock-in mice containing the Swedish double mutation of familial AD. As additional differences between mouse *APP* and human *APP* exon 16 were apparent, we also made nucleotide substitutions that encode for G676R, F681Y, and R684H to humanize the entire mouse exon 16 (Figure 1B). These mice were maintained as homozygous lines.

### Generation of NAIL mice

Homozygous NOG/APP^KM670/671NL^ (NA) mice were crossed with NOD. Cg-*Prkdc*^*scid*^*Il2rg*^*tm1Sug*^Tg(CMV*-IL34*)1/Jic (NOG/IL34) mice to generate heterozygous NOG/APP^KM670/671N^/IL34 (NAIL) mice. Heterozygous NAIL mice were backcrossed with homozygous NA mice to generate NAIL mice homogeneous for human *APP* exon 16 and heterogeneous for *IL34* gene. qPCR confirming the presence of the human IL-34 gene in NAIL mice (Figure 1C). We maintained the homozygous level of human *APP* exon 16 in NAIL mice to exclusively express only the human form of Aβ. The presence of human-Aβ in the NAIL mice was confirmed by positive 6E10 antibody staining. The intraneuronal expression of human Aβ in the cortex of NAIL mice was visualized in brain sections from NAIL and background NOG mice. Positive DAB staining was detected in HSC-reconstituted NAIL mice, with or without HIV-1 infection, confirming intraneuronal expression of human Aβ in NAIL mice. No staining was detected in NOG mice, indicating the absence of human Aβ in the mouse strain (Supplementary Figure 1).

### HIV-1 infection of NAIL mice and neuropathology

Neonatal (Day 2–3) NAIL mice were engrafted with CD34+ HSC (Figure 1D). By 3-months of age, engrafted NAIL mice were assessed for reconstitution of human immune cells in peripheral blood by flow cytometric analysis. NAIL mice were reconstituted with human CD45+, CD19+ (B cells), CD3+, CD4+, and CD8+ (T cells) as well as CD14+ monocytes (Figure 1E). Reconstituted NAIL mice were placed into infection studies with HIV_ADA_ by 4 months of age with plasma viral loads determined at days 10 and 25 post-infection and tissues dissected at day 25 for immunohistochemical and immunofluorescence examination (Figure 1D).

**Figure 2:**
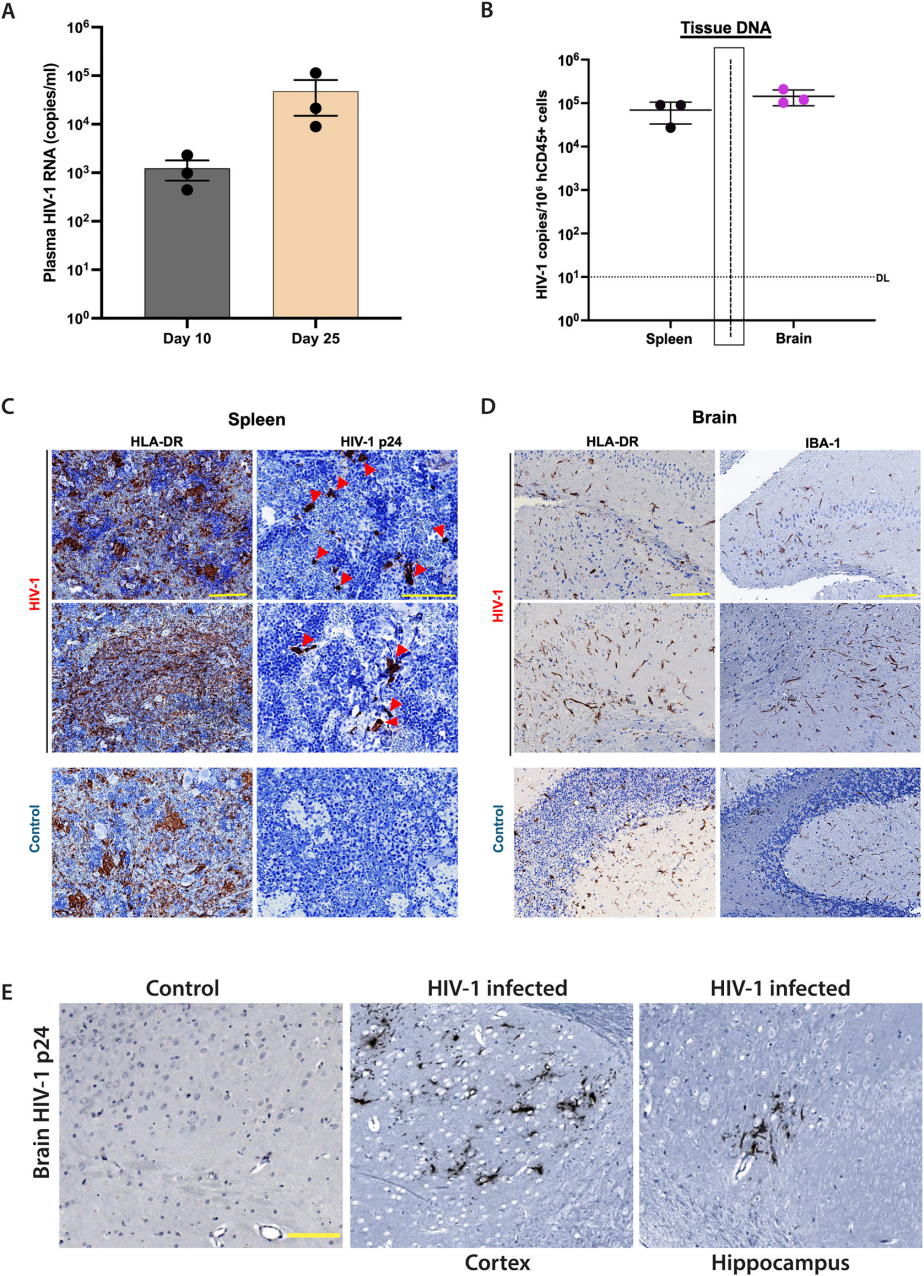
Immune-reconstituted NAIL mice support productive HIV-1 infection. NAIL mice were infected with 10^4^ TCID_50_ HIV-1_ADA_ and monitored for 25 days of infection before termination. HSC reconstituted NAIL mice treated with sterile saline served as uninfected controls. (A) Plasma HIV-1 RNA levels were determined on days 10 and 25 post-infection to measure viral load and disease progression. The detection limit (DL), 200 RNA copies/ml. (B) DNA was isolated from brain and spleen cells on day 25 post-infection. HIV-1 DNA levels were determined and normalized to the number of human CD45+ cells to indicate reservoir establishment after infection. Detection limit (DL), 10 DNA copies/10^6^ CD45+ cells. Tissue sections of spleen and brain were prepared from NAIL mice on day 25 post-infection or from uninfected controls. (C) Spleen sections were immunostained for expression of HLA-DR and HIVp24 (red arrowheads) to confirm productive HIV-1 infection in lymphoid tissues. (D) Brain sections were immunostained for HLA-DR and IBA-1 to validate the development of human microglia-like innate immune cells. (E) Immunohistochemical staining images of brain sections from control and HIV-1 infected NAIL mice stained for HIV-p24 protein. Positive HIV-p24 staining confirms productive viral infection in the brain. Scale bars, 100 μm.

Plasma HIV-1 RNA levels showed productive viral infection with an average RNA copy of 1.24 × 10^3^/ml at day 10 dpi and 4.81 × 10^4^/ml at 25 days after viral infection (Figure 2A). Viral DNA levels in tissues revealed HIV-1 DNA levels approximating 10^5^ copies/10^6^ human CD45+ cells in the spleen and brain by day 25 post-infection, thus indicating the establishment of HIV-1 reservoirs within the human immune cell populations in those tissues (Figure 2B). Spleen sections immunostained for HLA-DR and HIV-1p24 confirmed the presence HLA-DR + reconstitution of human immune cells that reside in the marginal zones of the spleen (Figure 2C). Moreover, numerous foci of HIVp24+ expressing cells were concomitantly found in spleens from infected NAIL mice, but not from uninfected NAIL controls. Brain sections stained for HLA-DR and IBA-1 showed similar expression patterns for IBA-1 and HLA-DR suggesting that IBA-1 reactive microglia are of human origin (Figure 2D). Moreover, substantially greater levels of both HLA-DR+ and IBA-1+ microglia were evident in brains from HIV-1 infected mice compared to uninfected controls. Immunohistochemical staining of brain sections stained for HIVp24 showed expression of the viral proteins in different brain sub-regions including the cortex and hippocampus from HIV-1 infected NAIL mice but not those from uninfected controls (Figure 2E).

**Figure 3:**
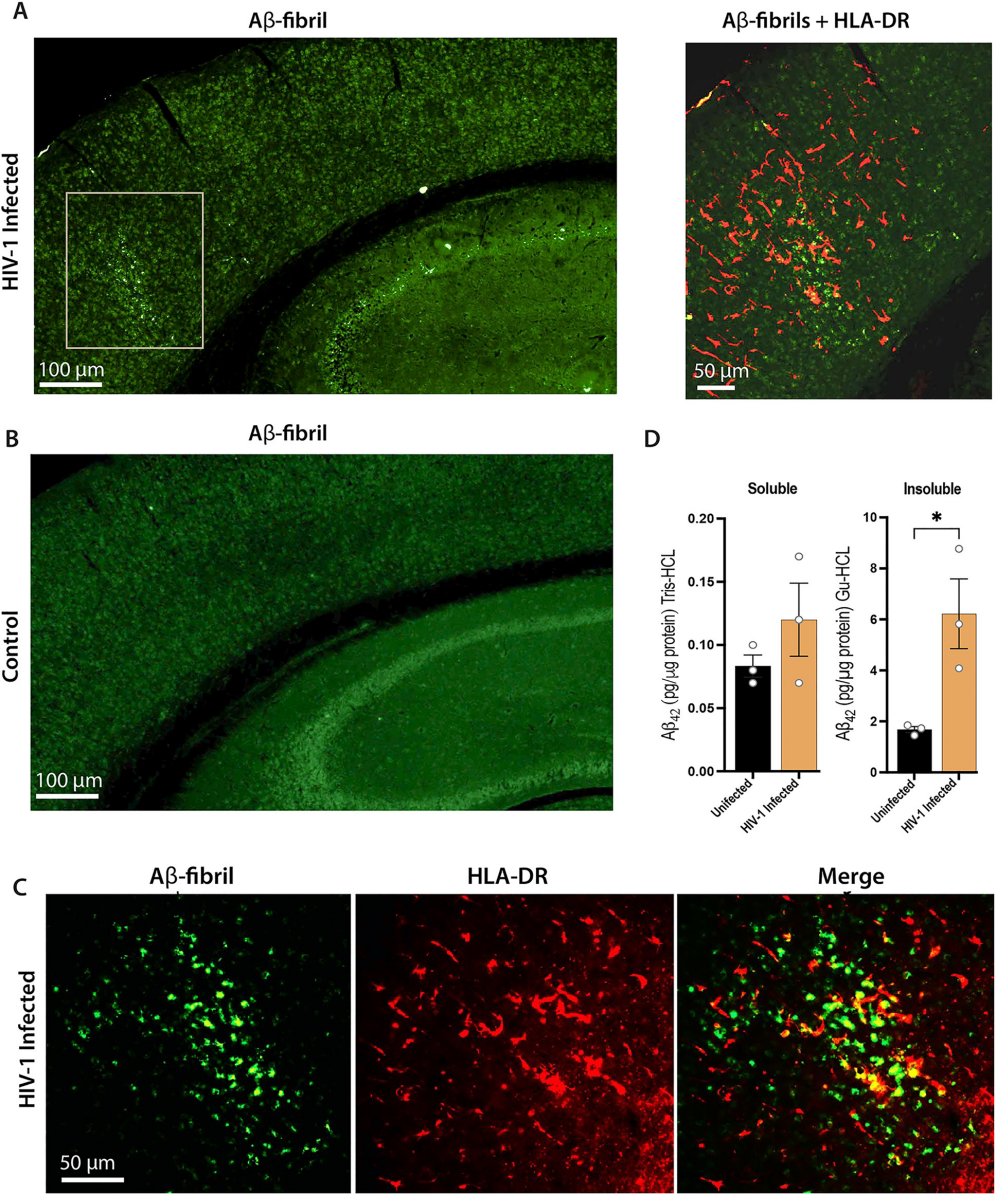
NAIL mice permit simultaneous measures of HIV-1 infection and AD pathobiology. NAIL mice were infected with 10^4^ TCID_50_ HIV-1_ADA_ or treated with saline as uninfected controls. Both groups were terminated at day 25 post-infection and sections of brain were evaluated by immunofluorescence. (A) Low (left panel) and high power magnification (right panel) of brain sections from HIV-1 infected NAIL mice stained for Aβ fibrils (OC antibody, green) denoted by gray box or both Aβ fibrils and HLA-DR (human immune cells, red) in the right panel. Scale bars, 100 μm (left panel) and 50 μm (right panel). (B) Brain sections from uninfected control stained for Aβ fibrils (OC antibody, green). Scale bar, 100 μm. (C) High power magnification of brain sections from HIV-1 infected NAIL mice stained for Aβ fibrils (OC antibody, green) and HLA-DR showing co-localization of human immune cells with Aβ-fibrils. Scale bar, 50 μm. (D) Cortical brain tissues from uninfected and day 25 HIV-1 infected NAIL mice were dissected, lysed, and fractionated by differential centrifugation. Soluble (Tris-HCl) and insoluble (Gu-HCl) lysate fractions were evaluated for Aβ_42_ levels by ELISA. Means ± SEM and significant differences were determined for three mice per group whereby *p≤0.05.

Brain sections from human immune-reconstituted NAIL mice stained for Aβ fibrils (OC+) showed numerous Aβ fibrils from HIV-1 infected NAIL mice (Figure 3A). Still, few fibrils were observed in the uninfected control mice (Figure 3B). Co-staining for OC+ Aβ fibrils and expression of HLA-DR showed co-localization of foci of Aβ fibrils with HLA-DR+ cells surrounded by a field of numerous HLA-DR + cells in brain sections from HIV-1 infected NAIL mice, but not from uninfected NAIL controls. Higher-power magnified sections of HIV-1 infected NAIL brains confirmed co-localization of Aβ fibrils with HLA-DR + cells in the brain (Figure 3C). To validate aggregated amyloid pathology, Aβ peptides in lysate fractions from cortical tissues of infected and control NAIL mice were assessed by ELISA. Levels of Aβ_42_ from insoluble lysate fractions of HIV-1 infected NAIL cortices were 3-fold more significant than those of uninfected NAIL controls. While soluble Aβ_42_ levels were higher in HIV-1 infected NAIL mice compared to uninfected controls but did not reach statistical significance (Figure 3D). Together, these findings are congruent with increased amyloidogenic pathology in HIV-1 infected NAIL mice compared to uninfected NAIL controls.

## Discussion

Herein, we developed a novel mouse model that serves, for the first time, to study the progression of AD in the setting of progressive HIV-1 infection. This was achieved through CRISPR-Cas9 knock-in of the familial human APP Swedish mutation (APP^KM670,671NL^) by substituting five nucleotides at exon 16 of the *APP* of NOG, thus generating NOG/APP^KM670,671NL^ (NA) mice. NA mice were crossed with the NOG mice transgenic for the human *IL34* gene to generate NOG/APP^KM670,671NL^/IL34 (NAIL) mice. These animals were reconstituted with human HSCs and developed innate and/or adaptive immune components in the periphery and central nervous system. The human *IL34* transgene in the NAIL mice enabled the development of “human-like” microglia in the brain following HSC engraftments [32].

In the present study, we performed a proof-of-concept study with six HSCs-engrafted NAIL mice. Three mice received 10^4^ TCID_50_ of HIV-1_ADA,_ and the remaining three received sterile saline as uninfected controls. The presence of human microglia-like cells in the brain facilitates HIV-1 infection [42]. Notably, HIV-1 infection of NAIL mice increased brain levels of soluble and insoluble Aβ; the latter was increased by 3-fold. Statistical differences were only observed in insoluble amyloid load but not in soluble amyloid load. Future studies are required with larger sample sizes, allowing a broader evaluation of the role of HIV-1 infection in driving AD pathology. Altogether, these findings of AD pathology in HIV-1-infected NAIL mice support the facilitation of increased amyloidogenic processes found in AD, like those reported in prior studies and HIV-1-infected human brains [16]. For the first time, the NAIL mouse model allows the analysis of crosstalk between human innate and adaptive immune cells in HIV infection and those associated with the pathology of misfolded AD proteins.

Several rodent models have been developed in the past to reflect HIV-1-associated neuropathogenesis, including transgenic mice expressing HIV-1 provirus and viral proteins as well as human immune cells-reconstituted mouse models [24], [43]. The first HIV-1 transgenic mouse model was developed by inserting full-length HIV-1 proviral DNA into the mouse genome under the control of a neurofilament promoter [44]. The model exhibited axonal degeneration with diminished motor activity and limb weakness. Similarly, other transgenic mice and rat models expressing HIV-1gag, gp120, or tat protein were developed to reflect neuronal injury and neuronal dysfunction associated with chronic HIV-1 infection in the CNS [43], 45], [46]. In humans, low levels of HIV-1 in the brain can elicit inflammatory responses by triggering the release of pro-inflammatory cytokines and chemokines, leading to neuronal injury and dysfunction. However, a low sustained level of viremia in the brain, as observed in humans, could not be replicated in transgenic rodent models, and hence, these models failed to recapitulate key HIV-associated comorbid complexities [43].

Human immune-reconstituted models that mimic HAND features have gained interest in recent decades [47], [48]. The first of a series of model systems induced HIV brain infections by injecting HIV-1 infected myeloid cells into the mouse brain that resulted in the development of HIV encephalitis (HIVE) [47]. While several critical features of viral infection were observed in the myeloid cell injection model, it was unable to recapitulate progressive disease, the spread of HIV-1 to brain subregions, or the response to ART [43]. Similarly, adult human peripheral blood lymphocyte (PBL) reconstitution models were developed to study the role of peripheral immune cells in driving HAND pathology, which was limited due to excessive host human-mouse cell reactions. Human PBL reconstituted mice exhibit short-lived engraftment but progressive HIV-1 infection with neuroinflammatory changes leading to increased murine amyloid and tau deposition in the brain [30]. However, the reconstitution of mice with adult human lymphocytes results in the rapid development of acute graft versus host disease (GvHD) [49], [50]. This has proven to be a significant limitation for utilization in the modeling of human HIV-1 pathogenic disease processes [29]. While prior mouse models have provided means for studying pathogenesis and drug efficacy, such reconstituted mice displayed signs of broad systemic inflammation that limited engraftment potential, which, combined with early mortality due to acute GvHD, precludes usefulness of those rodent models for studying HIV-1 mediated neuropathology [29], [43], [48], [51].

Human CD34+ HSC reconstituted models facilitate studies of progressive chronic HIV-1 infection. A severely immunodeficient mouse model such as NOG allows a higher level of stable immune cell engraftment [52], [53]. HIV infection is sustained for months in CD34+ HSC engrafted mice, making it suitable for testing therapies and studying long-term drug toxicities [40], [54]. However, this model also exhibits limitations, such as variation in the engraftment efficiencies of human cells, thus impacting neuropathological disease outcomes. Complex interactions of human pathological misfolded proteins such as Aβ, tau and α-synuclein with human immune cells in the context of HIV infection that contribute to neuropathogenesis cannot be studied in standard CD34+ HSC reconstituted models. Still, they must be studied in reconstituted models that provide sufficient capability for exploring those interactions [24], [43]. Due to its NOG background, the NAIL mouse model carrying a familial AD mutation permits human immune reconstitution. This makes it an ideal platform for studying other human-specific viruses, including herpes simplex virus, Epstein-Barr virus, and cytomegalovirus, which have been suggested to play a role in AD and related dementia [55], [56].

The current study developed the NAIL mouse line that incorporates a knock-in of the *APP* Swedish mutations associated with AD and carries a humanized Aβ sequence within an immunocompromised NOG background. This design enables human pathological Aβ protein expression by its endogenous promotor and amyloidogenic accumulation in the mouse brain. Following reconstitution with CD34+ HSCs, the *IL34* transgene in our NAIL mice facilitates the development of human microglia in the brain [32], [42]. Consequently, this model offers a unique opportunity to investigate the interactions between human peripheral immune cells and human myeloid cell responses to physiologically expressed human Aβ during progressive HIV-1 infection. This model will enable future studies to identify pharmacological targets for testing disease-modifying therapies for HAND, AD, and related dementias.

## Supplementary Material

Supplementary Material Details
