# Supplementary material for: HIV-1 infection facilitates Alzheimer’s disease pathology in humanized APP knock-in immunodeficient mice
Source: NeuroImmune Pharm Ther. 2025 Feb 10;4(1):27–38. doi: 10.1515/nipt-2024-0018 (PMC12041850; doi:10.1515/nipt-2024-0018)
Supplement: Supplementary file 1 — Supplementary Material Details [file j_nipt-2024-0018_suppl_001.docx]

**Supplementary figure 1**


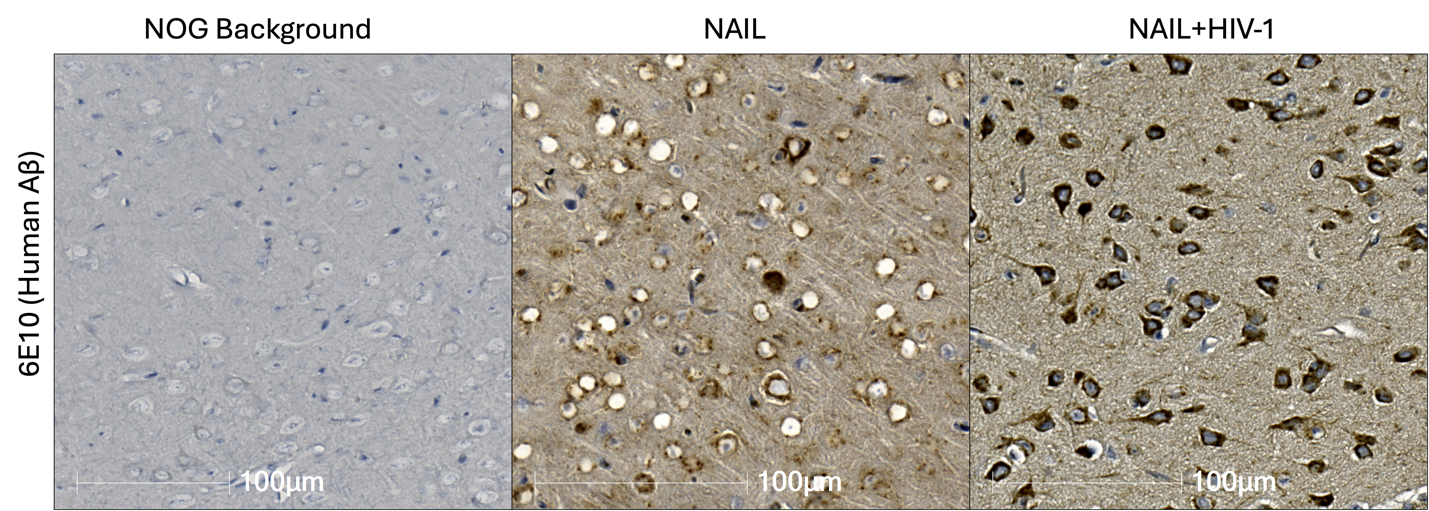


**Supplementary Figure 1**: **Intraneuronal expression of human Aβ in the cortex of NAIL mice.** 5 μm thick FFPE brain sections from NAIL mice and background NOG mice were stained with the human Aβ-specific antibody (6E10). Positive DAB staining was detected in HSC-reconstituted NAIL mice, with or without HIV-1 infection, confirming the intraneuronal expression of human Aβ in NAIL mice. In contrast, no staining was detected in the background NOG mice, indicating the absence of human Aβ in the background strain.
